# Effects of Quiet Eye Training on Performance of Bimanual Coordination in Children with DCD

**DOI:** 10.22037/ijcn.v15i4.18926

**Published:** 2021

**Authors:** Rasool Norouzi Seyed Hosseini, Ebrahim NOROUZI, Mehran Soleymani

**Affiliations:** 1 1.Department of Physical Education, Faculty of Humanities, Tarbiat Modares University, Tehran, Iran,; 2 Department of Motor Behavior, Faculty of Sport Sciences, Urmia University, Urmia, Iran,; 3 Department of psychology, Faculty of Education and Psychology, Azarbaijan Shahid Madani university, East Azarbaijan Province, Iran

**Keywords:** Developmental coordination disorder, Fine motor control, Visual feedback, Bimanual coordination, Quiet eye training

## Abstract

**Objectives:**

Children with developmental coordination disorder (DCD) are physically characterized by poor motor coordination and are at particular risk of losing their motor control. Quiet eye training (QET), with the latest techniques as an uncomplicated approach recently entered the field of rehabilitation research, has drawn the researchers’ attention. Therefore, the objective of this study was to examine the effects of QET on the performance of bimanual coordination in children with DCD.

**Materials & Methods:**

Children with DCD (n=20; aged 8-9 years) were randomly divided into two groups, including QET and traditional training (TT). The participants performed bimanual in-phase and anti-phase movements with their wrists at three speed levels ranging from slow to fast. Bimanual coordination accuracy was assessed at the baseline, after 4 weeks at study completion, and at the retention test.

**Results:**

Bimanual coordination improved over time from the baseline to study completion. The results showed that there was a significant difference between the pretest and posttest in the QET group (P=0.001), and bimanual coordination accuracy in the posttest significantly increased, compared to that reported for the pretest. Moreover, there was a significant difference between the pretest and posttest in bimanual coordination accuracy in the TT group (P=0.01), and the posttest accuracy significantly increased (F=2.32); however, the increase was less than that of the QET group.

**Conclusion:**

The obtained results indicated that the performance of the in-phase and anti-phase coordination modes was strongly influenced by QET. Furthermore, it was concluded that a successful performance of a bimanual linear task mainly depends on the availability of visual feedback.

## Introduction

Motor development is the process in which children suffuse movement patterns and motor skills (1). Normal childhood development follows a fairly predictable pattern; however, it sometimes causes problems created in the process of children’s motor development (2, 3). One of the disorders that affect motor coordination, gross motor skills, and fine motor skills is developmental coordination disorder (DCD). The condition is characterized by a poor performance of motor skills that has a significant and negative impact on daily activities (4). The DCD is used to describe children without neurological disorders or medical problems, particularly problems affecting academic and social functions (5). 

Studies related to children with DCD have shown that the use of various intervention programs has been noted in recent years. Zwicker et al. (2015) have shown that orientation tasks as an essential component of many interventions should be particularly regarded (6). Niemeijer et al. showed that motor function did not improve in children; nevertheless, intervention (i.e., physiotherapy exercises) has been effective (7). Over the past few years, the physiotherapy approach and occupational therapy of patients have evolved from traditional methods to modern and new methods, along with usual physiotherapy (8). Recently, new instruments, such as eye tracking, have gained many advantages in the treatment of motors disorders and motor control enhancement (9, 10). For example, quiet eye training (QET) with the latest techniques as an uncomplicated approach has recently entered the field of rehabilitation research (10, 11). 

The quiet eye (QE) was defined by Vickers (11) as the final fixation or tracking gaze on an object (for >100 MS to within 3° of visual angle) before the onset of a critical movement and has been observed to be a key predictor of perceptual-cognitive skill in a wide range of movement tasks. The QE has been shown to be a trainable key marker of proficient motor performance (10). The QET aims to help performers adopt the QE of a highly skilled prototype (10, 11). The initial studies of QET in the sporting domain have been successful in accelerating the novice performers’ skill acquisition, compared to traditional training (TT) (12). In this regard, Miles et al. (2014) conducted the first QET study, assessing the effectiveness of QET intervention in the improvement of performance in throwing and catching tasks. They observed that video-based QET intervention significantly improved catching performance by 23% in comparison to TT. Although the authors did not assess the longer-term impacts of QET in this population (i.e., at a delayed retention test, as opposed to immediately post-training retention test), the findings took a step forward in the determination of the transfer ability of QET to children suffering from DCD (13). 

Furthermore, a study by Wilson et al. (9) examined the QE and indicated that those with low motor coordination ability (<20^th^ percentile of Movement Assessment Battery for Children-2 [MABC-2] [14]) had significantly shorter QE durations during both throwing and catching tasks, compared to highly coordinated children (>70^th^ percentile of MABC-2). It was suggested that the longer QE duration prior to the throw helped to guide a more accurate throw which in turn helped them to more quickly locate the ball as it bounced off the wall. However, in the aforementioned study, the effect of QET has not been investigated (9).

Numerous daily life activities require continuous updating of the perception-action cycle to maintain the accuracy of human motor behavior (15). Among the different sensory modalities, vision has been identified to be essential for planning and guiding movements in time and space (16). For upper limb movements, the principles of coordination are realized in two stable patterns, namely in-phase (i.e., 0^0^ relative phases) and anti-phase (i.e., 180^0^ relative phase) (17-19). The in-phase coordination mode refers to mirror-symmetrical movements simultaneously made toward and away from the body midline (17, 20, 21). The anti-phase coordination mode refers to movements simultaneously made in the same direction from one side of the body midline to its different side, resulting in the performance of a parallel movement pattern in extrinsic space (20, 22, 23). 

At least for perceiving spatial information, vision dominates other senses (24). Many motor tasks are impossible or, at least, are much harder to perform without vision, such as walking on uneven terrain, hitting a tennis ball, or skiing (21, 24). Confirmed evidence that vision plays a critical role in the coupling of limb movements came from studies using both discrete (25) and cyclical bimanual movements (26). Specifically, these studies have shown that bimanual coordination movements were performed with higher levels of accuracy when visual information on the position of moving effectors was available, compared to those of other conditions where visual feedback was absent (27, 28). 

It is well established that predictive eye movements support the planning and controlling of goal-directed movements in natural environments (29). Furthermore, gaze behavior differentiates between children with and without DCD (30, 31). Accordingly, there are many QE discoveries yet to be made regarding motor tasks and for patients with disabilities and requiring rehabilitation (11). The gaze registration techniques provide an insight into how external visual information is used to guide and control goal-directed motor actions (30). Studies have shown that children with impaired motor coordination use less effective gaze strategies in controlled laboratory reaction time (32), visual tracking (30), and reach-to-grasp (33) tasks. Therefore, the current study aimed to examine the impacts of QET on the performance of bimanual coordination in children with DCD. 

## Materials & Methods

The participants were chosen based on the MABC test and were 20 elementary school children with DCD comprised of males aged 8 to 9 in Urmia, Iran. All the participants were undergraduate elementary school children. All the participants were right-handed (assessed by the Edinburgh Handedness Inventory [34]). The inclusion criteria were normal vision based on the Snellen chart test and self-reported normal audition. The exclusion criteria were 1) psychiatric problems, as ascertained by a brief psychiatric interview (Mini-International Neuropsychiatric Interview), 2) intake of mood- and arousal-medications or substances, 3) orthopedic problems, 4) uncorrected visual impairment, and 5) absence from educational sessions (more than two sessions). The children were novice to the bimanual coordination task and unaware of the study purpose. The parents completed a written informed consent form approved by the Local Ethics Board of Urmia University before the start of the study. 

Gaze behavior was recorded with a wireless SensoMotoric Instruments GmbH (SMI ETG 2w, Germany) using a mobile eye-tracking system consisting of glasses to track the eye movements and a controller to store the video recording. The SMI-ETG is a lightweight (47 g), wearable, and binocular system that uses dark pupil tracking to measure the point of gaze with a 60 Hz sampling rate and HD scene camera with a resolution of 1280 × 960p, 24 fps. 

The data from the eye tracker were recorded with a recording unit (i.e., Samsung Galaxy Note 4 smartphone, Korea) placed in a small waist bag worn by the examined participant. Three points calibration was conducted to verify the point-of-gaze before testing each study participant. Eye movements during a given gaze behavior were recorded using iWiewETG software (version 2.7). The data were analyzed frame-by-frame using SMI BeGaze software (version 3.6) installed on a Lenovo 80 23 laptop that was available for running remotely and observing or controlling the experiments.

**Figure 1:**
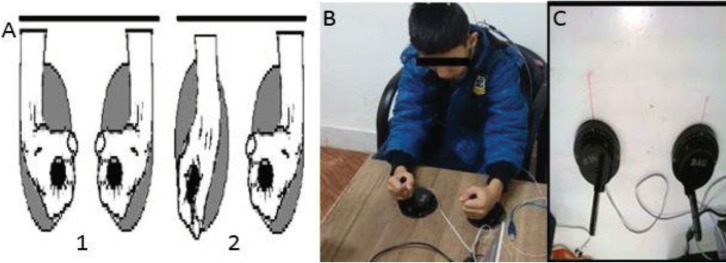
A) Grasping Two Handles and Moving Hands Together (1. In-phase and 2. Anti-phase Patterns); B) Participants Producing 180^0^ Relative Phase (Anti-phase); C) Bimanual Wrist Coordination Task

For the bimanual coordination assessment, the participants were sat on an adjustable chair at a table covered by a laminated poster board (50 cm deep and 86 cm wide). Wrist movements were permitted in only extension and flexion orientations from the midline (Figure 1). Attached in parallel to the slides were linear potentiometers (Bourns Instruments, Riverside, CA, United States), which encoded the displacement of the handled during a 20-second trial. An auditory metronome (i.e., NCH Swift Sound Tone Generator, version 2.01) provided pacing information for the bimanual tasks.

In the present study, the bimanual coordination task was used consisting of flexion and extension movements with both wrists in either an in-phase or anti-phase mode. The continuous nature of the bimanual actions in the present study required the participants to extensively control the limb in an online manner through visual feedback loops. The participants received a general orientation to the task. The task required them to grasp two handles attached to the moving slides and horizontally displace them in the left-right dimension. While grasping the two handles, the participants produced 0^0^ relative phases (in-phase) and 180^0^ relative phase (anti-phase) patterns. The data were sampled using a microprocessor (80486) with a sampling rate of 50 Hz (i.e., one sample each 5 ms). 

The smoothed position and velocity time series were then used to calculate each component of the near-continuous phase state for each trial according to the following formula adapted from Kelso et al. (18):

∅R=tan-1{(dXR/dt)/XR}

where ∅Ris the phase of the right wrist at each sample; *XR* is the position of the right wrist rescaled to the interval, {-1, 1} for each cycle of oscillation; (*dXR/dt*) is its normalized instantaneous velocity. The same formula was used to calculate the position and velocity signals of the left wrist. The relative phase () between the two wrists was then declared as follows:

  = ∅R-∅L

The participants were randomly divided into two groups after the pretest (i.e., TT and QET group). After the pretest, all the participants in both TT and QET groups were shown a video of the expert model performing the specific bimanual coordination training, overlaid with key visual prompts (a minute video recorded from an expert model in bimanual coordination). Both the TT and QET groups viewed the same video of a highly skilled movement performing the bimanual coordination task but with differing instructions. 

For the TT group videos, it was only shown a video of the perfect model performing bimanual coordination for a minute, and the children were asked to summarize this video to demonstrate their understanding. For the QET group videos, the expert model of performing bimanual coordination and gaze behavior demonstrating in the video was used to reveal the point of gaze of the expert model for the in-phase and anti-phase. The characteristics of the videos matched the instructions emphasized in the QET group. The QE measurement was based on the definition of Vickers (11) and Wilson et al. (9) as the final targeting fixation, with 1-3° of visual angle for a minimum of 100 ms, toward the wrists before the initiation of the movement (11, 35). The QE was analyzed in a frame-by-frame manner using BeGaze software (SensoMotoric Instruments, Germany).

The participants in both groups were met twice per week in small groups of 6 to 10 participants for 4 consecutive weeks (duration: 40 min per session). After the training, the participants performed posttest trials (i.e., the bimanual coordination task) while wearing the eye tracker system, but without any verbal feedback or guidance. The retention test was performed 48 h after the training session, just the same as the posttest (with the eye tracker and without feedback). 

Descriptive statistics, mean, and standard deviation were used in the present study. The normality of the data was checked, and age, intelligence quotient (IQ) scores, height, and weight of the participants were compared between the two groups via t-tests. Next, the analysis of variance (ANOVA) for repeated measures was performed with the factors, namely time (i.e., baseline, study end, and retention), group (i.e., QET vs. TT), and absolute error of relative phase (AE) as dependent variables. Post-hoc analyses were performed using Bonferroni corrections for p-values. 

Due to deviations from sphericity, ANOVA was performed using Greenhouse-Geisser corrected degrees of freedom, although the original degrees of freedom were reported with the relevant Greenhouse-Geisser epsilon value (ε). Since the unique features of QET were present in statistics, any possible change in each individual’s performance was individually checked due to any interference. The SPSS software (version 22) was used for comparing the calculations. The α-level of significance was set to 0.05.

## Results

There were no differences between the TT and QET groups in age (mean: 8.45 years; standard deviation (SD): 1.67); t [df, 2]: 0.453; P=0.64), mean total IQ (mean: 108.15; SD: 15.32); t [df, 2]: 0.567; P=0.54), height (mean: 127.6 cm; SD: 5.87 cm; t [df, 2]: 1.06; P=0.35), and weight (mean: 26.6 kg; SD: 4.21 kg; t [df, 2]: 1.03; P=0.19)}. 

The results of the 3 × 2 ANOVA for the AE scores (Table 1) revealed significant main effects for group (F [1]=7.79; P=0.01) and time (F [2]=47.98; P=0.001). The interaction effect for group and time was also significant (F [2]=6.052; P=0. 01). The significant two-way interaction for group × time was further analyzed using the Bonferroni posthoc test. Pairwise comparisons revealed a significant decrease of AE scores between the pretest to posttest (P=0.001), posttest to retention test (P=0.04), and pretest to retention test (P=0.05). Overall, these observations indicated that the performance of the in-phase and anti-phase coordination modes was strongly influenced by QET.

**Figure 2:**
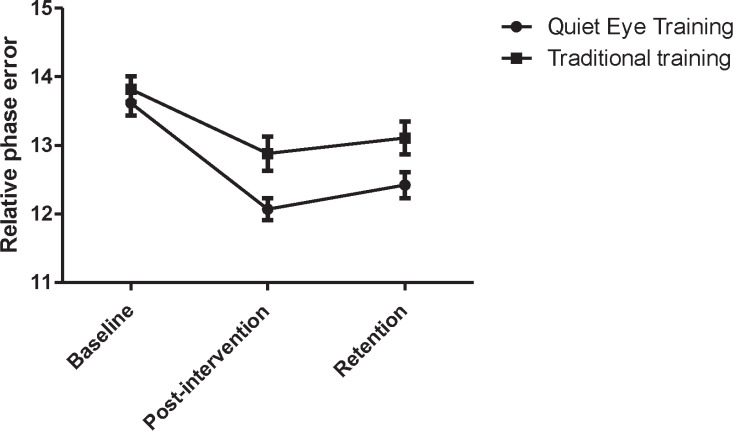
Mean and Standard Deviation of Relative Phase Errors in Different Groups and Test Stages

To further explore the significant group × test stage, one-way ANOVA of time was separately conducted for each group. Overall, one-way ANOVA revealed a significant effect of time for both two groups. As shown in Figure 2, regarding the accuracy coordination scores, there is a significant difference between the pretest and posttest in the QET group (P=0.001), and bimanual coordination accuracy in the posttest phase significantly increases, compared to that reported for the pretest (F=5.66). Moreover, there was a significant difference between the pretest and posttest in bimanual coordination accuracy in the TT group (P=0.01), and posttest scores significantly increased (F=2.32); however, the increase was less than that of the QET group.

**Table 1:** Descriptive Statistics for Bimanual Coordination, Separately for Groups and Measurement Points, and Tests for Time × Group Interaction Effects

**Groups**			**Factors**					
	Quiet eye training	Traditional training	Group		Time		Time × Group interaction	
***n***	20	20						
	Mean (Standard deviation)	Mean (Standard deviation)	*F*	η_p_^2^	*F*	η_p_^2^	*F*	η_p_^2^
Relative phase error			7.79*	0.46	47.98**	0.65	6.05*	0.40
Pretest	13.42 (1.13)	13.82 (0.99)						
Post-intervention Retention	12.07 (0.82) 12.42 (0.92)	12.88 (0.85) 13.11 (0.80)						

Notes: Degrees of freedom: Time: (2), Group: (1), Time × Group (2)

*P<0.05; **P<0.01

## Discussion

The experiment was designed to investigate the effects of QET on the performance of in-phase and anti-phase patterns of bimanual coordination in children with DCD. The obtained results indicated that visual feedback was more essential for a successful performance of the bimanual task. Overall, this finding revealed that when the children with DCD used QET, they performed both the in-phase and anti-phase coordination tasks with lower mean relative phase error, compared to TT when visual feedback of eye tracking was not presented. Stated differently, the results suggest that bimanual coordination movements appear to be controlled, for the main part, by visual information. Therefore, QET focused on helping the performer maintain control over key components of their visual attention and visuomotor coordination prior to and during performing the bimanual coordination task. These findings are primarily supported by the observations from previous studies where QET was examined (12, 13, 35-37). 

The present findings are in agreement with the observations reported in studies performed by Bourdin et al. (28), Vine et al. (35), Miles et al. (13), and Norouzi et al. (37), showing that the presence of visual information and QET enabled stable motor control. Moreover, the present results are in line with the results of Adolphe et al. (38), investigating the efficacy of QET in the improvement of motor skills. Furthermore, the results of the present study are consistent with the results of a study conducted by Vine and Wilson (12), demonstrating that QET resulted in longer QE durations and higher motor control. However, the present results are not consistent with the results of Norouzi et al. (39), showing that the performance of a bimanual linear coordination task mainly depends on the availability of proprioceptive input. In general, it is believed that the present results importantly add to the current literature showing that QET can improve bimanual coordination learning in children with DCD.

The availability of gaze behavior information affects motor performance. As such, in the present study, QET was successful in the improvement of bimanual coordination accuracy over the training stages, compared to TT (Figure 2). The latter finding suggests that visual monitoring influenced the production of both coordination modes in a different way. The interpretation is that during performing the bimanual coordination task, the extremities of both hands are in central vision when the reversal occurs in a flexed position. This position may allow for an adequate calibration in terms of spatiotemporal dimensions (26). 

The mechanisms behind the above-mentioned change in performance may be explained in relation to Land’s conceptualization of several distinct but interacting brain systems involved in the visual control of the action. Land suggests that the temporal and spatial relationships between gaze fixations and the action facilitate reflecting how top-down schema instructions are executed (11, 35). Therefore, by focusing on the desired location, the gaze system can forward information to the motor system, guiding the coordination of the requisite motor plans for the successful completion of the task (10, 11, 36). Furthermore, longer QE periods may also permit an extended duration of programming of these target parameters while minimizing possible distractions from irrelevant environmental cues (10).

The QET influenced the performance of the 180^0^ anti-phase and 0^0^ in-phase patterns. An explanation of these findings has been derived from the ideomotor theories of motor control (40). The crucial assumption of this approach is that motor actions are cognitively represented through their sensory effects, that is, with the codes of the perceptual effects contingently following certain motor actions. As a consequence, motor control can only be accessed by recollecting the codes of sensory consequences normally accompanying this action and serving to mentally represent it. Stated differently, there is no other way to generate a motor action but with anticipating its sensory consequences. 

The important implication of this approach is that all the constraints of motor control, such as complexity effects, stimulus-response compatibility, and/or symmetry tendencies, in bimanual coordination do not arise due to constraints inherent in the structure and/or functions of the motor system, but due to constraints in the representation of the perceptual re-afferences of to-be-produced motor actions (41). More studies should be conducted to further examine this hypothesis.

## Conclusion

The findings suggest that in children with DCD, QET in comparison to TT has the potential to enhance bimanual coordination, thereby contributing to the improvement of the motor control of children with DCD. To the best of our knowledge, this has been the first study to comprehensively examine the effects of QET on bimanual coordination in elementary school children with DCD. Therefore, there are some limitations and several future directions to be highlighted in the present investigation. Firstly, it appears that there was a ceiling effect on some outcome measures for children with DCD, including the stereotype. Further studies are required to extend the difficulty of these tests and determine the level at which children with DCD are challenged with these skills in functional tasks. Secondly, a larger number of participants may have led to significant outcomes for QET in elementary school children with DCD.
